# Serum and blood based biomarkers for lung cancer screening: a systematic review

**DOI:** 10.1186/s12885-018-4024-3

**Published:** 2018-02-13

**Authors:** Gavin C. W. Chu, Kim Lazare, Frank Sullivan

**Affiliations:** 1 Toronto Western Hospital Family Health Team, Department of Family and Community Medicine, University of Toronto, 2W428, 399 Bathurst Street, Toronto, ON M5T 2S8 Canada; 2 North York General Hospital Family Medicine Teaching Unit, Department of Family and Community Medicine, University of Toronto, 4 South, 4001 Leslie Street, Toronto, ON M6H 2Z7 Canada; 3 Department of Family and Community Medicine, University of Toronto, 500 University Avenue, 5th Floor, Room 348, Toronto, ON M5G 1V7 Canada; 4 Division of Population & Behavioural Sciences, Medical School, University of St Andrews, North Haugh, St Andrews, KY16 9TF UK

**Keywords:** Lung cancer, Screening, Systematic review, Biomarkers, Primary health care

## Abstract

**Background:**

Lung cancer is the second most common cancer and the leading cause of cancer death for both men and women. Although low-dose CT (LDCT) is recommended for lung cancer screening in high-risk populations and may decrease lung cancer mortality, there is a need to improve the accuracy of lung cancer screening to decrease over-diagnosis and morbidity. Blood and serum-based biomarkers, including EarlyCDT-lung and microRNA based biomarkers, are promising adjuncts to LDCT in lung cancer screening.

We evaluated the diagnostic performance of EarlyCDT-lung, micro-RNA signature classifier (MSC), and miR-test, and their impact on lung cancer-related mortality and all-cause mortality.

**Methods:**

References were identified using searches of PubMed, EMBASE, and Ovid Medline® from January 2000 to November 2015. Phase three or greater studies in the English language evaluating the diagnostic performance of EarlyCDT-lung, MSC, and miR-test were selected for inclusion.

**Results:**

Three phase 3 studies were identified, one evaluating EarlyCDT-lung, one evaluating miR-Test, and one evaluating MSC respectively. No phase 4 or 5 studies were identified. All three biomarker assays show promise for the detection of lung cancer. MSC shows promise when used in conjunction with LDCT for lung cancer detection, achieving a positive likelihood ratio of 18.6 if both LDCT and MSC are positive, and a negative likelihood ratio of 0.03 if both LDCT and MSC are negative. However, there is a paucity of high-quality studies that can guide clinical implementation.

**Conclusions:**

There is currently no high quality evidence to support or guide the implementation of these biomarkers in clinical practice. Reports of further research at stages four and five for these, and other promising methods, is required.

**Electronic supplementary material:**

The online version of this article (10.1186/s12885-018-4024-3) contains supplementary material, which is available to authorized users.

## Background

Lung cancer is the second most common cancer and the leading cause of cancer death for both men and women [1–3]. In 2015, an estimated 26,600 Canadians were diagnosed with, and 20,900 died from, lung cancer [2]. In 2014, 163,422 patients from the UK died from lung cancer, with lung cancer projected to continue as the leading cause of cancer-related death until 2035 [3]. The five year survival rate for patients diagnosed with late stage lung cancer and metastatic lung cancer are 16.8% and < 5% respectively [1]. Conversely, the 5-year survival rate of small intrapulmonary cancers is 80% [4]. Therefore, identification of lung cancer at an early stage could potentially lead to significant decreases in morbidity and mortality [5, 6].

In 2010, the National Lung Screening Trial (NLST) demonstrated a 20% reduction in lung cancer mortality and 7% reduction in all-cause mortality by screening patients at high risk of lung cancer with low-dose chest CT (LDCT) scans (NNS = 320) [1, 4]. However, 24.2% of patients who had LDCT exhibited abnormal findings, and 96.4% of these findings were false positive results, representing a 18% over-diagnosis rate [1, 4, 7]. The high rate of false positives has led to multiple screening rounds with high radiation exposure, a high use of harmful diagnostic follow-up, increased patient costs and anxiety [4, 8]. Therefore, while LDCT may be effective in reducing lung cancer mortality, there is a need to improve the accuracy of lung cancer screening to decrease morbidity and health-care associated costs.

Molecular biomarkers are potentially useful adjuncts to LDCT for lung cancer screening, either by further delineating patient risk prior to LDCT, or assessing malignant risk of positive LDCT findings [1, 4, 6, 9, 10]. The performance of any test also depends upon the prior probability of the condition in the population being sampled and this varies considerably [11, 12]. Biomarkers may be generated from cancer cells, the tumor microenvironment, or the host response to cancer [4, 13]. Various molecular factors that are implicated in lung carcinogenesis have been evaluated as prognostic and diagnostic biomarkers, such as markers of apoptosis, cellular adhesion, cellular growth, and tumor proliferation [10, 14]. Epigenetic markers such as DNA methylation, miRNAs, nucleosome remodeling, and histone modifications have also been investigated [10, 13, 14]. Biomarkers may be sampled from many different bodily sources, including whole blood, serum, plasma, bronchial brushings, and sputum [13, 14]. Circulating blood-based and serum-based biomarkers are a convenient compartment to sample as they are relatively easy and inexpensive to collect [4, 6, 9].

The EarlyCDT-Lung test is a commercially available blood test based on ELISA principles that measures a panel of seven tumor-associated autoantibodies: p53, NY-ESO-1, CAGE, GBU4–5, SOX2, HuD, and MAGE A4 [15]. The miR-test is a serum based miRNA test that measures a signature of 13 miRNAs: miR-92a-3p, miR-30b-5p, miR-191-5p, miR-484, miR-328-3p,miR-30c-5p, miR-374a-5p, let-7d-5p, miR-331-3p, miR-29a-3p, miR-148a-3p, miR-223-3p, miR-140-5p [16]. The MSC is a plasma-based miRNA test that categorizes patients into low, intermediate, or high risk of disease based on pre-defined positivity for 24 miRNA expression ratios [17]. Of the available blood and serum-based biomarkers, only EarlyCDT-Lung, Serum-based miRNA signature (miR-test), and Plasma-based miRNA test (MSC) have entered Phase 4 of development [4]. There is, therefore, a need to evaluate the current state of biomarker development, especially EarlyCDT-Lung and miRNA based strategies, to guide future research in lung-cancer screening.

This literature review describes the diagnostic performance of EarlyCDT-Lung, miR-test, and MSC as adjunctive biomarkers to LDCT for the diagnosis of lung cancer. The key questions considered for this review are:

1. What is the individual diagnostic performance of each of EarlyCDT-Lung, miR-test, and MSC for the detection of lung cancer?
2. What is the diagnostic performance of EarlyCDT-Lung, miR-test, and MSC used in conjunction with LDCT for the detection of lung cancer?
3. Does screening with EarlyCDT-Lung, miR-test, and MSC with or without LDCT improve lung-cancer mortality and all-cause mortality?

## Methods

### Search strategy

A literature review was conducted at North York General Hospital, a suburban academic teaching hospital in Toronto, Canada. We searched Ovid MEDLINE ®, EMBASE, and PUBMED from 2000 up to November 2015 for any lung cancer diagnostic trials involving EarlyCDT-Lung, miR-test, and MSC published in English. We also checked reference lists of included studies and relevant systematic reviews. The full search strategy is available in Additional file 1: Appendix 1.

### Study selection

After removing duplicates, all citations titles were evaluated for relevance utilizing inclusion criteria for this review. Citation titles were evaluated independently by GS and FS, with consensus amongst both PIs required for inclusion. Articles marked for inclusion by either team member went on to abstract relevance testing. Abstract screening was done independently by GS and FS, with consensus required for inclusion or exclusion.

### Inclusion and exclusion criteria

#### Language

The published results of studies had to be available in English.

#### Population

The population of interest for the review was asymptomatic adults age 18 and older who were at high risk but were not suspected of having lung cancer. Patients known to have lung cancer or were previously diagnosed with lung cancer were excluded from the study population.

#### Interventions

The three screening interventions of interest were:

1. EarlyCDT-Lung, an antibody based biomarker screening panel
2. miR-test, a serum-based 13 miRNA signature
3. micro-RNA signature classifier (MSC), a plasma-based 24 miRNA risk score

#### Study design

To answer the key questions, only Phase 3 or Phase 4 studies that included one or more measures of diagnostic performance (sensitivity, specificity, likelihood ratio, etc) in the abstract were included. All studies that were Phase 1, Phase 2, or did not mention any diagnostic performance measure were excluded.

#### Outcomes

The key outcomes evaluated in this review included:

1. Diagnostic performance for detection of lung cancer
2. Outcome performance in reducing lung cancer-related mortality and all-cause mortality

### Data abstraction and assessment of study validity

For each included study, we extracted data about the population, study design, intervention, inclusion criteria, exclusion criteria, the analysis, and results for the outcome of interest. To evaluate for validity and bias, each included study was evaluated against the STARD 2015 checklist [18]. Any concerns regarding bias or the validity of the study was recorded in the data collection template. The full data collection template is available in Additional file 1: Appendix 2.

## Results

### Summary of literature search

**Fig. 1:**
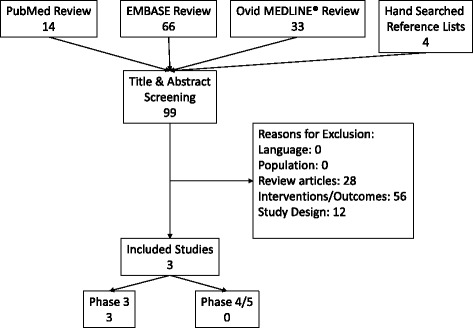
Search and selection results

Our search for studies examining the diagnostic and outcome performance of EarlyCDT-lung, miR-test, and MSC with and without LDCT located 99 unique citations (Fig. 1). From these searches, we identified 28 review articles on the topic of biomarkers for lung-cancer screening. On-topic non-review studies were identified for abstract screening. 12 of the remaining 15 studies were excluded for being Phase 1 or 2 trials and did not meet the inclusion criterion of Phase 3 and above. 56 studies were excluded because the paper described interventions or outcome which used biomarkers and 12 were excluded because the study design did not enable the calculation of test performance characteristics.

The reference lists of included studies and identified review articles were examined, but no additional studies that met inclusion criteria were identified. Therefore, 3 identified studies met inclusion criteria and were included in the review.

### Summary of included studies

Three phase 3 studies evaluated the diagnostic performance of various blood-based biomarkers for lung cancer detection, one evaluating EarlyCDT-lung [19], one evaluating miR-Test [16], and one evaluating MSC [17] respectively. No phase 4 or 5 studies were identified. Although all three studies were phase 3 studies, inclusion criteria and study design differed significantly. A summary of study characteristics is available in Table 1. A full summary of each included study is available in Additional file 1: Appendix 3.

**Table 1:** Summary of study characteristics

	Jett et al. 2014	Sozzi et al. 2014	Montani et al. 2015
Test Evaluated	EarlyCDT-lung	MSC	miR-test
Number of patients	1613	939	1008
Patient Inclusion Criteria	EarlyCDT-lung test made available to treating physicians Clear inclusion criteria not defined	MILD trial participants: > 20 pack-years smoking history > 50 years old without history of cancer in past 5 years. 1000 consecutive plasma samples from trial participants Additional 69 plasma samples from 85 patients with lung cancer in MILD trial	COSMOS trial participants: > 20 pack-years smoking > 50 years old Lung cancer patients diagnosed outside of COSMOS trial
Patient Exclusion Criteria	Clear exclusion criteria not defined	Hemolyzed samples No known pulmonary pathology	No known pulmonary pathology
Follow-up Period	6 months	5 years	Unknown
Key Study limitations	Audit trial used in regular physician practice No clear eligibility criteria No clear lung cancer diagnostic criteria No baseline characteristics of population No distribution of alternative diagnosis in those without target condition No discussion of study limitations, biases, uncertainty No link to full study protocol No discussion of sources of funding	No discussion of how sample size was determined No distribution of alternative diagnosis for those without lung cancer	No indication of whether clinical information available to performers/readers of tests No discussion of how sample size was determined No distribution of alternative diagnosis in those not diagnosed with lung cancer Very brief discussion of study limitations

Jett et al. evaluated the use and diagnostic performance of EarlyCDT-lung in 1613 patients presenting to 810 unique physicians in 720 different practices in 48 states [19]. The EarlyCDT-Lung test was offered to patients at the discretion of the treating physician. Clear inclusion/exclusion criteria for whom to offer the test were not stated. The definition for a positive screening result included any antigen titration series showing a dose response and one or more auto-antibodies resulting above the previously validated clinical cut-off. Patients were followed for a period of 6 months and the treating physician decided on a lung cancer diagnosis. Confirmation by an external lung cancer expert was sought if evidence challenging the diagnosis was found.

Sozzi et al. evaluated the diagnostic performance of the MSC in 1000 consecutive plasma samples from 4099 participants enrolled in the Multicenter Italian Lung Detection (MILD) Trial [17]. The MILD trial was a RCT involving 4099 current or former smokers of greater than 20 pack-years and at least 50 years of age without history of cancer in the past year, evaluating the effectiveness of LDCT for lung cancer screening; 2376 enrolled patients were randomly assigned to the LDCT arms and 1723 to the observation arm [20]. 130 of the 1000 plasma samples collected were excluded due to hemolysis. 69 samples from the 85 patients identified with lung cancer in the entire MILD trial were included, resulting in a total number of 939 plasma samples. Patients were followed for a period of 5 years as part of the MILD trial.

Montani et al. evaluated the diagnostic performance of the miR-test in a “validation set” of 1008 patients enrolled in the Continuous Observation of Smoking Subjects (COSMOS) trial and lung cancer patients diagnosed outside of the screening trial [16]. The COSMOS trial is an ongoing observational trial evaluating LDCT screening in patients greater than 50 years old with a greater than 20 pack-year smoking history and without any diagnosed pulmonary pathology [21]. 1008 individuals enrolled in the COSMOS study including 36 patients with low-dose computed tomography (LDCT)-detected lung cancer and 972 individuals without lung cancer, randomly selected from the entire COSMOS consecutive cohort from March 2011 to March 2012 were included in this study.

### Diagnostic performance for detection of lung cancer

The diagnostic performance of each biomarker test used alone for the detection of lung cancer is summarized in Table 2. EarlyCDT-lung showed a sensitivity, specificity, PPV, NPV, and positive likelihood ratio of 41%, 87%, 11%, 97%, and 3.19 respectively. MSC had a sensitivity, specificity, PPV, NPV, and positive likelihood ratio of 87%, 81%, 27%, 98%, and 4.67 respectively. miR-test had a sensitivity, specificity, PPV, NPV, and positive likelihood ratio of 78%, 75%, 10%, 98%, and 3.09 respectively.

**Table 2:** Diagnostic performance of biomarkers alone for detection of lung cancer

Test Evaluated	EarlyCDT-lung	MSC	miR-test
Sensitivity	41% (95% CI: 29–53%)	87% (95%CI: N/A)	78% (95%CI: N/A)
Specificity	87% (95% CI: 86–89%)	81% (95% CI: 79–84%)	75% (95% CI: 72–78%)
PPV	11% (95% CI: 7–15%)	27% (95% CI: 21–32%)	10% (95% CI: 7–14%)
NPV	97% (95% CI: 97–98%)	98% (95% CI: N/A)	98% (95% CI: N/A)
Positive LR	3.19	4.67	3.09
Negative LR	0.68	0.16	0.30

The MSC was evaluated for its diagnostic performance in conjunction with LDCT. If positive results for both MSC and LDCT were needed for a positive screen, a sensitivity, specificity, PPV, NPV, and positive likelihood ratio of 69%, 96%, 65%, 97%, and 18.6 was achieved. If only one of MSC or LDCT needed to be positive to result in a positive screen, a sensitivity, specificity, PPV, NPV, and negative likelihood ratio of 98%, 66%, 22%, 99%, and 0.03 was achieved.

### Lung cancer-related mortality and all-cause mortality

miR-test and MSC were evaluated for the important outcome of lung-cancer related mortality (Table 3). The miR-test had a sensitivity, specificity, PPV, NPV, and positive likelihood ratio of 100%, 73%, 1%, 100%, and 3.72 respectively for lung cancer-related mortality. The MSC had a sensitivity, specificity, PPV, NPV, and positive likelihood ratio of 95%, 78%, 8%, 99%, and 4.27 respectively for lung cancer-related mortality. There were a total of 3 lung-cancer deaths in the study evaluating miR-test and 19 lung-cancer deaths in the study evaluating MSC.

**Table 3:** Diagnostic performance of biomarkers alone for lung-cancer death

Test Evaluated	MSC	miR-test
Sensitivity	95% (95%CI: N/A)	100% (95%CI: N/A)
Specificity	78% (95% CI: 75–80%)	73% (95% CI: 70–76%)
PPV	8% (95% CI: 5–12%)	1.1% (95% CI: N/A)
NPV	99% (95% CI: N/A)	100% (95% CI: N/A)
Positive LR	4.27	3.72

The MSC was evaluated for overall mortality. However, no death occurred due to other causes in lung cancer–free participants.

## Discussion

EarlyCDT-lung, miR-test, and MSC were chosen as the focus for this review as they are reported as the biomarkers at the most advanced phase of development for the detection of lung cancer [4]. This review focuses on clinically relevant measures for lung cancer screening, including measures of diagnostic performance and impact on lung cancer-related mortality and all-cause mortality.

All three biomarkers show promise in their diagnostic ability to detect lung cancer. The plasma-based micro-RNA signature classifier (MSC) trended towards the highest sensitivity, specificity, and positive likelihood ratio for the detection of lung cancer. However, a direct comparison between the three biomarker signatures cannot be made at this time as sample sizes are small, confidence intervals for performance measures are wide, no trials have directly compared the three biomarker signatures, and the number of trials evaluating each biomarker is singular.

The only trial that directly evaluated the diagnostic ability of a blood-based biomarker in conjunction with LDCT shows promise that biomarkers can be useful adjuncts to LDCT in screening for lung cancer. When using MSC in conjunction with LDCT, a positive likelihood ratio of 18.6 was achieved if both MSC and LDCT were positive, while a negative likelihood ratio of 0.03 was achieved if both MSC and LDCT were negative. This suggests that biomarker signatures may potentially be a means to risk stratify at-risk patients for the development of lung cancer.

Although blood-based biomarkers show promise, there currently is no high quality prospective literature to guide the implementation of blood-based biomarkers in clinical practice for lung cancer detection. Prospective phase 4 studies are currently ongoing to assess the value of the above biomarkers for their value as a pre-CT screening tool. The Early Lung Cancer Detection Study (ECLS) is currently ongoing in Scotland, randomizing approximately 12,000 people from the Greater Glasgow and Clyde area to the EarlyCDT-lung test or routine care (ClinicalTrials.gov ID: NCT01925625) [22]. Patients with a positive Early-CDT-lung test undergo a CT scan at baseline followed by CT scans every 6 months for 24 months. The primary outcome is the difference at 24 months in the number of patients with late stage lung cancer (Stages 3 and 4). The COSMOS II study enrolling approximately 10,000 high risk subjects in Italy will evaluate prospectively miR-Test in conjunction with LDCT [16]. Similarly, the Plasma microRNA Profiling as First Line Screening Test for Lung Cancer Detection (BIOMILD) trial will enroll approximately 4000 subjects to evaluate the MSC as a potential first line screening test for lung cancer (ClinicalTrials.gov ID: NCT02247453) [23].

Limitations of this work include the small number of studies identified and the substantial variability across studies in terms of inclusion criteria, methodology, follow-up, timing, and comparators. The number of patients enrolled was small and follow up period for each study was relatively short. It is important to note that the inclusion criteria for these studies varied regarding pack-years of smoking and how patients were enrolled from their larger parent trials. These trials were conducted in different countries where attitudes, laws, and public health policies regarding smoking differed. As our search focused on the three biomarker signatures (EarlyCDT-Lung, miR-test, and MSC), studies regarding other biomarker signatures would not have been included. Finally, studies published in languages apart from English would not have been included.

## Conclusions

Although blood and serum-based biomarkers are promising adjuncts to LDCT for the detection lung cancer, there is currently no high quality evidence to support or guide the implementation of these biomarkers in clinical practice. Prospective studies are ongoing to evaluate the diagnostic performance and impact of biomarkers on clinically relevant outcomes. Further research is required to guide clinical implementation.

## Additional file

Additional file 1: **Appendix 1** - Search strategy; **Appendix 2** - Data collection template; **Appendix 3** -Summary of included studies. (DOC 146 kb)

## Abbreviations

COSMOS: Continuous Observation of Smoking Subjects trial
LDCT: Low-dose computer tomography
LR-: Negative likelihood ratio
LR +: Positive likelihood ratio
MILD: Multicenter Italian Lung Detection trial
miRNA: micro-RNA
miR-test: serum-based 13 miRNA signature
MSC: micro-RNA signature classifier
NLST: National Lung Screening Trial
NPV: Negative predictive value.
PPV: Positive predictive value.
