# Supplementary material for: Serum and blood based biomarkers for lung cancer screening: a systematic review
Source: BMC Cancer. 2018 Feb 13;18:181. doi: 10.1186/s12885-018-4024-3 (PMC5812229; doi:10.1186/s12885-018-4024-3)
Supplement: Supplementary file 1 — Appendix 1 - Search strategy; Appendix 2 - Data collection template; Appendix 3 -Summary of included studies. (DOC 146 kb) [file 12885_2018_4024_MOESM1_ESM.doc]

## Appendix 1: Search Strategy

Search Strategy:

--------------------------------------------------------------------------------

1 exp *Lung Neoplasms/

2 "Early Detection of Cancer"/

3 "earlyCDT-lung".mp.

4 "miRNA".ti,ab.

5 ("serum miRNA" or "plasma miRNA").mp.

6 3 or 5

7 low-dose CT lung cancer screening.mp.

8 exp Mass Screening/

9 exp Tumor Markers, Biological/ or exp MicroRNAs/ or exp Biological Markers/

10 "34 miRNA".mp.

11 "plasma-based miRNA test".mp.

12 "MSC miRNA test".mp.

13 "MicroRNA signatures".mp.

14 "plasma-based miRNA".mp.

15 exp Carcinoma, Small Cell/ or exp Carcinoma, Non-Small-Cell Lung/ or exp Carcinoma, Lewis Lung/ or exp Carcinoma, Squamous Cell/

16 1 or 15

17 MicroRNAs/

18 6 or 10 or 13 or 14 or 17

19 16 and 18

20 9 and 19

21 3 or 7

22 20 and 21

23 limit 20 to yr="2000 -Current"

24 2 and 23

25 from 24 keep 4-6,8-9,11-31

26 10 and 16

27 ("low dose CT" or "LDCT").mp.

28 18 and 27

29 from 28 keep 1,3-4

30 25 or 29

31 8 and 9 and 19

32 30 or 31

33 8 and 18 and 27

34 32 or 33

## Appendix 2: Data Collection Template

| Title |  |
| --- | --- |
| Year |  |
| Authors |  |
| Journal Name |  |
|  |  |
| Study phase |  |
| Number of patients/controls |  |
| Patient Inclusion Criteria |  |
| Patient Exclusion Criteria |  |
| Screening tool(s) investigated |  |
| Criteria for “positive” screening result |  |
| Patient characteristics |  |
|  |  |
| **Diagnostic Performance (Test alone)** |  |
| Sensitivity |  |
| Specificity |  |
| PPV |  |
| NPV |  |
| Odds Ratio |  |
| Lung-cancer mortality |  |
| All-cause mortality |  |
|  |  |
| **Diagnostic Performance (Test + LDCT)** |  |
| Sensitivity |  |
| Specificity |  |
| PPV |  |
| NPV |  |
| Odds Ratio |  |
| Lung-cancer mortality |  |
| All-cause mortality |  |
|  |  |
| **Validity** |  |
| Study Limitations/Concerns |  |

## Appendix 3: Summary of Included Studies

| **Title** | **Audit of the autoantibody test, EarlyCDT-Lung, in 1600 patients: An evaluation of its performance in routine clinical practice** |
| --- | --- |
| Year | 2014 |
| Authors | Jett, J. R.;L. J. Peek;L. Fredericks;W. Jewell;W. W. Pingleton, et al. |
| Journal Name | Lung Cancer |
|  |  |
| Study phase | 3 |
| Number of patients/controls | 1613 |
| Patient Inclusion Criteria | Patients presenting to physician offices  810 unique physicians in 720 different practices in 48 US states |
| Patient Exclusion Criteria | N/A |
| Screening tool(s) investigated | EarlyCDT-Lung 6AAB panel (n=752)  EarlyCDT-Lung 7AAB panel (n=861) |
| Criteria for lung cancer diagnosis | Clinical according to treating physician  Confirmation by external expert if evidence challenging Dx |
| Criteria for “positive” screening result | Antigen titration series showed dose response and one or more AAB level above clinical cut-off |
| Patient characteristics | Not stated |
| Time of follow up | 6 months |
|  |  |
| **Diagnostic Performance (Test alone for lung cancer diagnosis)** |  |
| Sensitivity | 25/61, 41% (95% CI: 29-53%) |
| Specificity | 1341/1538, 87% (95% CI: 86-89%) |
| PPV | 11% (95% CI: 7-15%) |
| NPV | 97% (95% CI: 97-98%) |
| Positive LR | 3.19 |
| Negative LR | 0.68 |
|  |  |
| **Diagnostic Performance (Test alone for lung cancer mortality)** | Not assessed |
|  |  |
| **Diagnostic Performance (Test + LDCT)** | Not assessed |
|  |  |
| **Validity** |  |
| Study Limitations/Concerns | Audit trial used in regular physician practice  No clear eligibility criteria  No clear lung cancer diagnostic criteria  No sample size calculation  No baseline characteristics of population  No distribution of alternative diagnosis in those without target condition  No discussion of study limitations, biases, uncertainty  No link to full study protocol  No discussion of sources of funding |

| **Title** | **Clinical Utility of a Plasma-Based miRNA Signature Classifier Within Computer Tomography Lung Cancer Screening: A Correlative MILD Trial Study** |
| --- | --- |
| Year | 2014 |
| Authors | Sozzi, G.;M. Boeri;M. Rossi;C. Verri;P. Suatoni, et al. |
| Journal Name | Journal of Clinical Oncology |
|  |  |
| Study phase | 3 |
| Number of patients/controls | 939 |
| Patient Inclusion Criteria | MILD Trial participants: 4099 current or former smokers of at least 50 years of age without history of cancer in past 5 years. 2376 (58%) assigned to LDCT (1190 annual, 1186 biennial) and 1723 (42%) to observation arm.  No lung cancer: 1000 consecutive plasma samples from trial collected from trial participants  Lung cancer: 69 of 85 with patient with lung cancer had valid plasma samples |
| Patient Exclusion Criteria | 130 excluded due to hemolysis in sample |
| Screening tool(s) investigated | MSC algorithm |
| Criteria for “positive” screening result | 24miRNAs detected  Used to classify patients into low, intermediate, or high risk  Positive = Intermediate and High risk |
| Patient characteristics | Lung cancer patients older (60.9 vs. 56.4, p<0.01)  Lung cancer patients more male (81.2% vs 63.3%, p=0.003)  Lung cancer patients smoked for longer |
|  |  |
| **Diagnostic Performance (Test alone for lung cancer detection)** |  |
| Sensitivity | 60/69, 87% (95%CI: N/A) |
| Specificity | 708/870, 81% (95% CI: 79-84%) |
| PPV | 27% (95% CI: 21-32%) |
| NPV | 98% (95% CI: N/A) |
| Positive LR | 4.67 |
| Negative LR | 0.16 |
|  |  |
| **Diagnostic Performance (Test alone for lung cancer mortality)** |  |
| Sensitivity | 18/19, 95% (95%CI: N/A) |
| Specificity | 716/920, 78% (95% CI: 75-81%) |
| PPV | 8% (95% CI: 5-12%) |
| NPV | 99% (95% CI: N/A) |
| Positive LR | 4.27 |
| Negative LR | 0.06 |
|  |  |
| **Diagnostic Performance (Test AND LDCT for lung cancer diagnosis)** |  |
| Sensitivity | 40/58, 69% (95% CI: 57-81%) |
| Specificity | 572/594, 96% (95% CI: 95-98%) |
| PPV | 65% (95% CI: 53-76%) |
| NPV | 97% (95% CI: 96-98%) |
| Positive LR | 18.6 |
| Negative LR | 0.32 |
|  |  |
| **Diagnostic Performance (Test OR LDCT for lung cancer diagnosis)** |  |
| Sensitivity | 57/58, 98% (95% CI: N/A) |
| Specificity | 199/594, 66% (95% CI: 63-70%) |
| PPV | 22% (95% CI: 17-27%) |
| NPV | 99% (95% CI: N/A) |
| Positive LR | 2.93 |
| Negative LR | 0.03 |
|  |  |
| **Validity** |  |
| Study Limitations/Concerns | No discussion of how sample size was determined  No distribution of alternative diagnosis for those without lung cancer |

| **Title** | **miR-Test: A Blood Test for Lung Cancer Early Detection** |
| --- | --- |
| Year | 2015 |
| Authors | Montani, F.;M. J. Marzi;F. Dezi;E. Dama;R. M. Carletti, et al. |
| Journal Name | JNCI J Natl Cancer Inst |
|  |  |
| Study phase | 3 |
| Number of patients/controls | 1008 |
| Patient Inclusion Criteria | COSMOS trial participants: heavy smokers, older than 50 years old  Lung cancer patients diagnosed outside of screening |
| Patient Exclusion Criteria | Not stated |
| Screening tool(s) investigated | miR-test |
| Criteria for “positive” screening result | 13miRNA signature evaluated using qRT-PCR  miR-test risk score calculated using in-house automated script  Risk score >0 is positive, <0 negative |
| Patient characteristics | No significant differences in age, gender, smoking status (Supplemental Table 1) |
|  |  |
| **Diagnostic Performance (Test alone for lung cancer detection)** |  |
| Sensitivity | 28/36, 78% (95%CI: N/A) |
| Specificity | 727/972, 75% (95% CI: 72-78%) |
| PPV | 10% (95% CI: 7-14%) |
| NPV | 98% (95% CI: N/A) |
| Positive LR | 3.09 |
| Negative LR | 0.30 |
|  |  |
| **Diagnostic Performance (Test alone for lung cancer mortality)** |  |
| Sensitivity | 3/3, 100% (95%CI: N/A) |
| Specificity | 735/1005, 73% (95% CI: 70-76%) |
| PPV | 1.1% (95% CI: N/A) |
| NPV | 100% (95% CI: N/A) |
| Positive LR | 3.72 |
|  |  |
| **Diagnostic Performance (Test AND LDCT for lung cancer diagnosis)** | Not enough information to assess |
|  |  |
| **Diagnostic Performance (Test OR LDCT for lung cancer diagnosis)** | Not enough information to assess |
|  |  |
| **Validity** |  |
| Study Limitations/Concerns | No indication of whether clinical information available to performers/readers of tests  No discussion of how sample size was determined  No distribution of alternative diagnosis in those not diagnosed with lung cancer  Very brief discussion of study limitations only |
